# Expected Benefits From Antibacterial Envelope in Different CIED Patient Populations

**DOI:** 10.1111/pace.70038

**Published:** 2025-09-16

**Authors:** David Duncker, Christine Alonso, Mauro Biffi, Francisco Costa, Rim El Bouazzaoui, Panagiota Flevari, Maria Hee Jung Park Frausing, Andreas Goette, Didier Klug, Edward Maclean, Tobias Tönnis, Emilce Trucco, Andrew J. Turley, Jay Wright, Bruno Valente, Matteo Ziacchi

**Affiliations:** ^1^ Department of Cardiology and Angiology Hannover Heart Rhythm Center Hannover Medical School Hannover Germany; ^2^ GHP Ambroise Paré‐Hartman Neuilly sur Seine France; ^3^ Institute of Cardiology, IRCCS Azienda Ospedaliero‐Universitaria di Bologna Bologna Italy; ^4^ Hospital da Luz, SA Lisbon Portugal; ^5^ Centre Hospitalier Libourne Libourne France; ^6^ Attikon University Hospital Athens Greece; ^7^ Aarhus University Hospital Aarhus Denmark; ^8^ St. Vincenz Hospital, Paderborn, Germany and MAESTRIA Consortium/AFNET Münster Germany; ^9^ University of Lille Lille France; ^10^ Bartholomew's Hospital London UK; ^11^ University Heart and Vascular Center Hamburg Germany; ^12^ Hospital Universitari de Girona Dr Josep Trueta Girona Spain; ^13^ James Cook University Hospital Middlesbrough UK; ^14^ Liverpool Heart and Chest Hospital Liverpool UK; ^15^ Centro Hospitalar Universitário de Lisboa Central Lisbon Portugal

**Keywords:** antibacterial envelope, CIED implantation, CIED infection, pocket healing

## Abstract

Antibacterial envelope has proven to be safe and effective in reducing cardiac implantable electronic device (CIED) infection. Recently, its role in pocket healing process has also started to be studied, showing additional potential benefits for the patients. Risk scores such as the PADIT and the BLISTER ones have been developed to stratify patients at higher risk of developing CIED infections, but their use is still limited. There is a need for clear and concise practical guidance and scores for the correct stratification of patients at risk to develop CIED infections, to apply an effective preventive strategy. This article examines the most recent findings on the effectiveness of antibacterial envelopes in preventing CIED infections and introduces the latest concept of pocket stabilization. Additionally, patient populations that could potentially benefit from antibacterial envelope for both indications are provided based on the agreement of an expert panel made up of 16 European electrophysiologists from high‐volume centers. Future studies will have to evaluate the strength of those proposals and the benefits of pocket stabilization in the long‐term.

## Graphical abstract

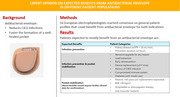

**Abbreviations**
BLISTER: The acronym “BLISTER” stands for different clinical factors that contribute to the overall score: Blood markers, Lead extraction, Immunocompromised status, Surgery duration, Type of procedure, EGFR, Re‐intervention within 2 years
BMI: body mass index
CIED: cardiac implantable electronic device
CRP: C‐reactive protein
CRT: cardiac resynchronization therapy
CRT‐D: cardiac resynchronization therapy with defibrillator
EGFR: estimated glomerular filtration rate
ICD: implantable cardiac defibrillator
PADIT: Prevention of Arrhythmia Device Infection Trial
WHO: World Health Organization

## 1 Introduction

In recent years, a rise in infections related to cardiac implantable electronic devices (CIEDs) has been recorded, continuing to cause high rates of mortality and morbidity even with the best available treatments [1, 2, 3]. The significant impact on patient outcomes and quality of life, as well as on healthcare costs, requires a comprehensive commitment from the healthcare community to minimize infections [2].

The WRAP‐IT randomized controlled trial showed that using a fully resorbable antibacterial envelope (TYRX, Medtronic Inc., Minneapolis, MN) during device re‐interventions or CRT‐D implants significantly reduced the risk of infection at 12 months and this technology has been incorporated into the EHRA consensus document for high‐risk patients [4, 5, 6]. However, the definition of high‐risk patients is broad, and budget constraints in healthcare make it challenging to strictly follow the recommendations [3]. Risk scores such as the PADIT score [7] and BLISTER score [3] have been developed and validated to identify patients at higher infection risk and allow a cost‐effective use of the antibacterial envelope. Nevertheless, CIED infection predictive scores are rarely used in clinical practice for different reasons, including lack of knowledge, perceived low reliability and complexity [2].

**What's new?**

There is a need for clear and concise practical guidance for the correct stratification of patients at risk of developing CIED infections, to apply an effective preventive strategy. The antibacterial envelope has shown a significant role in CIED infection prevention, when used in the right patients. In addition, its role in pocket healing may have benefits beyond infection, especially in patients undergoing multiple secondary procedures.

An expert panel, made up of 16 European electrophysiologists from high‐volume centers, agreed on general patient populations that could potentially benefit from antibacterial envelope for both CIED infection prevention and pocket stabilization indications.

The CIED pocket is the host tissue around the implanted CIED that gradually transforms into a fibrotic capsule [8, 9]. Inflammation, along with the resulting scarring, thickening, and calcification of the device capsule, can complicate access to the pocket during device replacement and increase the risk of complications [8, 10, 11]. Therefore, it is important to minimize these effects: the concept of achieving a healthy pocket that is less fibrotic, has better vascularization, and is more prone to bleeding, while remaining durable over time, is starting to be known as “pocket stabilization”. This approach aims to create an optimal environment for the device, reducing complications and improving long‐term outcomes. The second‐generation absorbable antibacterial envelope was designed to wrap an implanted CIED, providing a framework that supports the formation of a stable, thin, fibrous capsule. The TYRX Pocket Health Study recently showed low adhesions, low inflammation, and well formed capsules in patients with implanted antibacterial envelope undergoing device replacement after an average of 6.8 years [10].

A recently published European survey on CIED infection awareness highlighted the need for clear and concise practical guidance and scores for the correct stratification of patients at risk to develop CIED infections, to apply an effective preventive strategy [2].

The aim of this review is to share a practical advice, based on available literature, experts’ opinions, and experiences on which patient populations could benefit more from an antibacterial envelope for both CIED infection prevention and pocket stabilization.

### 1.1 Antibacterial Envelope and Other CIED Infection Prevention Strategies

**FIGURE 1:**
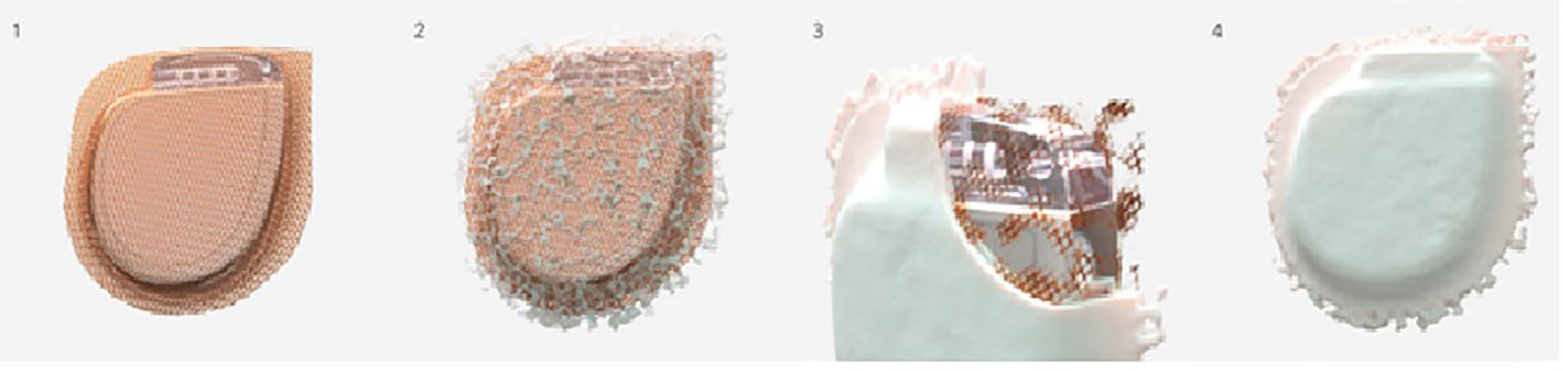
Pocket stabilization concept: phases of fibrous capsule formation around a CIED enclosed in an antibacterial envelope. (1) Phase 1–Antibacterial Envelope Wrapping a CIED: antibacterial envelope encasing a cardiac implantable electronic device (CIED). (2) Phase 2–Initial Fibrous Capsule Development: early stages of fibrous capsule formation around the antibacterial envelope. This phase marks the beginning of the body's response to the implanted device, with connective tissue starting to grow and surround the envelope. (3) Phase 3–Advanced Capsule Formation: the fibrous capsule is nearing completion around the antibacterial envelope. The connective tissue has significantly developed, almost entirely covering the envelope and providing a stable foundation. (4) Phase 4–Complete Capsule Formation: fully developed fibrous capsule surrounding the CIED within the antibacterial envelope. This mature capsule creates a stable and secure interface between the tissue and the envelope, ensuring long‐term stability and integration of the device. [Colour figure can be viewed at wileyonlinelibrary.com]

Antibacterial envelopes are fully absorbable and can be used for CIED, including ICDs, pacemakers, and neurostimulators (Figure 1). The surgical mesh envelope is gradually absorbed by the body over approximately 9 weeks. During this period, it integrates with the surrounding tissue, creating a stable and secure environment for the device. Containing the antibiotics minocycline and rifampin, it is intended to stabilize the device by providing a physical structure that holds it in place, preventing it from shifting or moving within the pocket, and reducing the risk of infection [12, 13]. Infections occur in about 1%–4% of all CIED implantations, resulting in a 14% mortality rate within the first year and up to a 50% mortality rate within 3 years [14, 15, 16, 17]. The average treatment cost is 36,722€ [18].

The TYRX envelope (Medtronic Inc., Minneapolis, MN) has demonstrated a 40%–60% reduction of infections among high‐risk patients [6, 19, 20, 21]. It also reduces the chance of device migration, erosion, or Twiddler's syndrome [19]. In Europe, this approach has been shown to be cost‐effective [22].

With the growing number of patients with comorbidities receiving CIEDs, along with an increase in revision, upgrade, and replacement procedures, infections are rising at a faster rate than CIED implantations [18]. A recently published survey involving more than 300 European centers showed that preoperative antibiotic prophylaxis is routinely administered to all patients, while the tissue pocket is irrigated with antibiotic agents perioperatively, and oral antibiotics are prescribed after the procedure only in around 20% of centers [2]. Other strategies to prevent CIED infections are the implant of the lowest number of leads, according to patient's implant indication, and the implant of leadless devices [2]. An antibacterial envelope is used by 57% of respondents, but only in high‐risk patients [2]. High‐risk patients are selected in around 50% of cases by medical opinion, whereas in the remaining 50% a risk score is used. The most commonly used score is the PADIT score, based on five independent variables: prior procedure(s), age *<* 70 years, depressed renal function, immunocompromised status, and ICD, CRT or revision/upgrade procedures (Table 1) [7]. Recently, the BLISTER score has been developed to increment the prognostic value of the PADIT score by adding significant covariates: lead extraction, C‐reactive protein > 50 mg/L, re‐intervention within 2 years, and procedure duration (Table 1) [3]. The BLISTER score demonstrated superior discriminative performance versus PADIT in all patients [3].

**TABLE 1:** PADIT [7] and BLISTER [3] scores comparison.

Score component	Criteria	PADIT points	BLISTER points
Prior procedure	1 prior CIED procedure	1	2
	≥ 2 prior CIED procedures	3	4
Age	< 60	2	2
	60–69	1	1
	≥ 70 years	0	0
Depressed renal function/Blood results	eGFR < 30 mL/min	1	2
	CRP ≥ 50 mg/L	0	1
Immunocompromised*	Receiving therapy that suppresses resistance to infection or having a disease that is sufficiently advanced to suppress resistance to infection	3	2
Procedure type	ICD	2	1
	ICD generator change	NA	1
	CRT	4	4
	CRT generator change	NA	4
	Revision/Upgrade	4	NA
	New lead inserted or existing lead revised	NA	4
	Lead extracted	NA	6
Long procedure time	Pocket open for ≥ 120 min during procedure	0	2
Early re‐intervention	Intervention on the same pocket within 2 years of a previous procedure	0	7

*Note*: PADIT score considers patients with ≤ 4 points at low risk and patients with ≥ 7 points at high risk. BLISTER score recommends the use of antibacterial envelope in patients with ≥ 6 points. For the PADIT score, all these items are included under the “Revision/Upgrade” criteria, whereas for the BLISTER score, different points are assigned depending on the type of Revision/Upgrade.

Abbreviation: NA, not applicable.

While both PADIT and BLISTER score could be helpful in selecting high‐risk patients that would derive the greatest clinical benefit from the antibacterial envelope, these tools should be part of a wider strategy to preventing CIED infection.

The WHO Surgical Safety Checklist [22] is an effective tool to identify high‐risk patients; infection risk should be discussed for every case in the preoperatory phase.

### 1.2 Pocket Stabilization

Pocket stabilization is a relatively new concept with only preliminary data available. However, through this review, we aim to raise awareness within the scientific community about this emerging idea.

The implantation of a CIED triggers a foreign body reaction, resulting in encapsulating fibrosis that stabilizes the device within the tissue [8]. The specifics of the implant procedure, along with the type of device and leads used, significantly influence the nature of tissue remodeling. This resulting capsule forms a stable, thin, fibrous barrier between the CIED and the host tissue [8]. However, excessive movement of the implanted device or prolonged inflammation in the pocket can lead to thickening or calcification of the capsule. Fibrotic and calcified capsules complicate pocket revision procedures, raising the risks of infection, hematoma formation, and lead fractures [11, 23]. A recent large animal study compared the TYRX antibacterial envelope (Medtronic Inc., Minneapolis, MN) against the non‐antibacterial CanGaroo extracellular matrix envelope (Aziyo Biologics, Inc., Silver Spring, MD) and no envelope at all (control) [8]. The study results indicate that early healing processes at the tissue–envelope interface help stabilize the CIED. TYRX envelope pockets exhibited lower levels of inflammation, quicker provisional matrix formation, faster absorption, and thinner capsules. TYRX envelope pockets demonstrated similar inflammatory and healing profiles to controls, with more rapid provisional matrix formation compared to both controls and CanGaroo extracellular matrix envelope. CanGaroo extracellular matrix envelope pockets showed increased acute inflammation at 3 and 7 days, and chronic inflammation at 24 weeks. TYRX envelop pockets were almost completely absorbed by week 12, whereas CanGaroo extracellular matrix envelope remained present at week 24 and was associated with significantly thicker capsules [8].

Given the critical role of capsule formation in protecting the device throughout its lifetime and during subsequent revisions, antimicrobial envelope may promote an accelerated healing process and an earlier establishment of a stable device environment (Figure 1). The TYRX Pocket Health Study showed that pocket stabilization effects are persistent at long‐term follow‐up (7 years) [10]. There is no evidence on which patients could benefit most from a long‐term pocket stabilization: robust scientific evidence will be required to validate the advantages of this approach that is currently not reported in the scientific guidelines. Additionally, future investigations should focus on well designed clinical studies to evaluate the benefits of pocket stabilization in diverse patient populations, with the aim of identifying subgroups that might benefit the most from this approach.

## 2 Methods

### 2.1 Expert Panel Composition and Panel Meetings

The 16 members of the Expert panel from high‐volume centers in Europe were selected based on their experience with antibacterial envelopes and participation in the project including one face‐to‐face and one online panel meeting.

The experts shared their opinion about the benefit of implanting antibacterial envelope in different patient populations for two indications: (1) CIED infection prevention and (2) pocket stabilization.

To reach a consensus, the following steps were followed:

- An online survey was completed by the Panel to evaluate their strategy to prevent CIED infections in different patient populations. Prior to the start of the project, it was decided that agreement was considered reached when > 75% of responders strongly agreed, agreed, or were neutral.
- After an in‐person meeting to discuss the survey results, a second survey round was completed to reach consensus on the different patient populations that could benefit more from antibacterial envelope for the two above indications.

The project was funded by Medtronic, but the company did not influence the statements by the expert. panel.

## 3 Results

### 3.1 Expert Panel Agreement on the Benefits of an Antibacterial Envelope

A summary of the expected benefits of antibacterial envelope in different patient populations can be found in Table 2. A summary of the agreement results for each survey statement is provided in Table 3.

**TABLE 2:** Expected benefits of antibacterial envelope in different patient populations.

Expected benefits	Patient categories
Infection prevention	Kidney disease (eGFR < 30 mL/min)
	Procedure duration ≥ 2 h
Infection prevention & pocket stabilization	Revision/upgrade for any reason
	Lead extraction
	Early re‐intervention
	Device replacement (≥ 2nd time)
	History of CIED infection
	Compromised immune system
	Diabetes (with insulin use)
Pocket stabilization *(these benefits would require further data for confirmation)*	Patients < 60 years (≥ 2 replacements expected)
	BMI > 40 kg/m^2^
	Antiplatelet or anticoagulant therapy
	Submuscular implants

**TABLE 3:** Agreement results for each survey statement.

Question	Agreement	Neutral	Disagreement
*Benefits are expected in the use of antibacterial envelope, independently of* ***device type*** *and of other risk factors*:			
**‐ In patients undergoing revision/upgrade**	**100%**	**0%**	**0%**
**‐ In patients undergoing device replacement for ≥ 2nd time**	**100%**	**0%**	**0%**
**‐ In patients undergoing lead extraction**	**81.3%**	**6.2%**	**12.5%**
**‐ In patients undergoing early re‐intervention**	**100%**	**0%**	**0%**
*Benefits are expected in the use of antibacterial envelope, independently of the* ***procedure type*** *and of other risk factors*:			
**‐ In patients with history of CIED infection**	**100%**	**0%**	**0%**
**‐ In patients with kidney disease (eGFR ≤ 30 mL/min)**	**68.8%**	**31.2%**	**0%**
**‐ In patients with procedure duration likely ≥ 2 h**	**68.8%**	**31.2%**	**0%**
**‐ In patients with diabetes with insulin use**	**75%**	**19%**	**6%**
**‐ In patients with compromised immune system**	**94%**	**6%**	**0%**
*Benefits are expected in the use of antibacterial envelope for* ***pocket stabilization*** *, independently of the procedure type and of other risk factors*:			
‐ In patients receiving anticoagulation	50%	38%	12%
‐ In patients receiving dual antiplatelet therapy	43.7%	43.8%	12.5%
**‐ In patients post‐implant hematoma requiring revision**	**88%**	**6%**	**6%**
**‐ In young patients expected to have ≥ 2 replacements in the future**	**56.3%**	**37.5%**	**6.2%**
‐ In patients with BMI < 16.5	37.4%	43.8%	18.8%
**‐ In patients with BMI > 40**	**56.3%**	**37.5%**	**6.2%**
‐ In patients with subpectoral device implant	44%	44%	12%
‐ In patients undergoing revision procedure due to Twiddler's syndrome	75%	25%	0%
**‐ In patients undergoing pacemaker replacements for ≥ 2nd time**	**88%**	**12%**	**0%**

*Note*: Statements for which agreement was considered reached are highlighted in bold.

### 3.2 Benefits Expected in the Use of Antibacterial Envelope for both CIED Infection Prevention and Pocket Stabilization

One hundred percent of the expert panel members agreed on the beneficial effect of antibacterial envelope for both CIED infection prevention and pocket stabilization in revision/upgrade, early re‐intervention procedures, and device replacement for ≥ 2nd time, independently of CIED device type and of any other risk factors. Revision and upgrades are considered significant risk factors in both PADIT and BLISTER score, whereas device replacement and early re‐intervention were only reported in BLISTER score (Table 1) [3, 7].

In the context of lead extraction procedures, 14 out of the 16 members (87.5%) of the expert panel highlighted the need for further research on the universal benefits of antibacterial envelopes for both CIED infection prevention and pocket stabilization. One member cited a lack of evidence, whereas the other argued that the benefit depends on the reason for extraction: specifically, if the extraction is due to infection and reimplantation occurs at a different site, the antibacterial envelope would not be advantageous. In many cases of device extraction, a leadless device is now implanted to reduce the risk of infection. Consequently, the use of an antibacterial envelope in the extracted pocket may not be necessary in these cases. All expert panel members (100%) agreed on the beneficial effect of antibacterial envelope for both CIED infection prevention and pocket stabilization in patients with previous history of CIED infection on the same implant side, independently of procedure type and of any other risk factors. The history of previous infections is not considered in either the PADIT score or BLISTER score (Table 1) [3, 7].

All expert panel members (100%) agreed on the beneficial effect of antibacterial envelope for both CIED infection prevention and pocket stabilization in patients with compromised immune system, defined as steroid or immunosuppressant medication or immunocompromised by comorbidity, for example, HIV infection, in agreement with both PADIT and BLISTER score definitions, but independently of CIED device type and of any other risk factors.

An agreement (75% agree and 19% neutral) on the beneficial effect of antibacterial envelope for both CIED infection prevention and pocket stabilization was also reached for patients with insulin‐treated diabetes, defined as being under insulin use, independently of CIED device type and of any other risk factors. This is not represented in any risk score, despite being highly considered as a risk factor in clinical practice by European implanting physicians [2].

### 3.3 Benefits Expected in the Use of an Antibacterial Envelope for CIED Infection Prevention

An agreement (69% agree and 31% neutral) on the beneficial effect of antibacterial envelope for CIED infection prevention for both patients with kidney disease (eGFR <30 mL/min) and procedure duration likely ≥ 2 h was reached, independently of CIED device type and of any other risk factors. Procedure duration is reported as risk factor only in the BLISTER score [3].

### 3.4 Benefits Expected in the Use of Antibacterial Envelope for Pocket Stabilization

An agreement (56.3% agree and 37.5% neutral) on the beneficial effect of antibacterial envelope for pocket stabilization was reached for patients < 60 years expected to have ≤ 2 replacements in the future and in obese patients, defined as having BMI > 40 kg/m^2^. For patients with a BMI below 16.5, there was no consensus reached (31.2% agree and 31.2% neutral): despite all expert panel members agreeing on the fact that very thin patients have a higher risk of developing pocket hematoma, the main concern was that using an antibacterial envelope in these patients could be challenging precisely due to the patient's thinness. An agreement on the beneficial effect of antibacterial envelope for pocket stabilization was reached for patients under anticoagulation (50% agreement and 38% neutral) or antiplatelet therapy (44% agreement and 44% neutral), and for patients undergoing subpectoral implants (44% agreement and 44% neutral). The main reason for disagreement in all the statements related to benefits expected in the use of antibacterial envelope for pocket stabilization was the lack of clinical evidence.

Subcutaneous pocket for endovascular ICD was also considered as a factor that could potentially benefit from an antibacterial envelope for pocket stabilization, although it was not included in the survey.

## 4 Conclusions

This review provides expert opinion–based advise on specific patient populations that could potentially benefit from an antibacterial envelope for both CIED infection prevention and pocket stabilization. Although there is already considerable clinical data supporting the indication for CIED, future studies will need to assess the strength of these proposals and the long‐term benefits of pocket stabilization.

## Author Contributions

All authors were involved in the development of the concept, answering the survey, participating in meetings, and reviewing the manuscript.

## Conflicts of Interest

D.D. received modest lecture honorary, travel grants, and/or a fellowship grant from Abbott, Astra Zeneca, Biotronik, Boehringer Ingelheim, Boston Scientific, Bristol Myers Squibb, CVRx, Medtronic, MicroPort, Pfizer, and Zoll. A.J.T. received modest lecture honorary, travel grants, and/or a fellowship grant from Abbott, Astra Zeneca, Boehringer Ingelheim, Bristol Myers Squibb, and Medtronic. M.Z. received speaker fees from Abbott, Biotronik, Boston Scientific, and Edwards Lifesciences. E.T. received modest lecture honorary, travel grants, and/or fellowship grant from Abbott, Biotronik, and Medtronic. All the other authors have no conflicts of interest.

## Data Availability

Data sharing is not applicable to this article as no new data were created or analyzed in this study.
